# Central auditory processing in children after traumatic brain injury

**DOI:** 10.1016/j.clinsp.2022.100118

**Published:** 2022-10-03

**Authors:** Carolina Calsolari Figueiredo de Godoy, Adriana Neves Andrade, Italo Suriano, Carla Gentile Matas, Daniela Gil

**Affiliations:** a Speech-Language-Hearing Pathologist, Universidade Federal de São Paulo, São Paulo, SP, Brazil; b Neurologist, Universidade Federal de São Paulo, São Paulo, SP, Brazil

**Keywords:** Hearing, Auditory perceptual disorders, Traumatic brain injury, Child, Hearing tests

## Abstract

**Introduction:**

Traumatic brain injury can impair the central auditory pathways and auditory cortex. Hence, individuals who suffered a traumatic brain injury may be at risk of central auditory processing disorders, which can be identified with behavioral tests that assess central auditory function.

**Objective:**

To characterize and compare the performance of children and adolescents with and without a history of traumatic brain injury in behavioral tests that assess central auditory processing.

**Method:**

The sample comprised 8- to 18-year-old individuals of both sexes who suffered moderate or severe closed traumatic brain injury 3 to 24 months before their participation in the study and whose hearing thresholds were normal. These individuals were matched for sex and age with other subjects without a history of traumatic brain injury and submitted to behavioral assessment of the central auditory processing with special tests to assess hearing skills (namely, auditory closure, figure-ground, and temporal processing), selected according to their chronological age and response-ability.

**Results:**

The study group performed statistically worse than the comparison group in auditory closure, figure-ground in verbal dichotic listening, and temporal ordering. The central auditory processing tests with abnormal results in the comparison group were different from those in the study group.

**Conclusion:**

Central auditory processing disorders were identified in all subjects of the study group, especially involving auditory closure and temporal processing skills, in comparison with subjects without a history of traumatic brain injury.

## Highlights

• Central auditory processing disorder was detected in all the individuals in the study group.
• The study group had worse performance with a statistically significant difference.
• The changes especially are in auditory closure and temporal processing as compared to the control group.

## Introduction

Traumatic Brain Injury (TBI) is any traumatic aggression resulting in an anatomical lesion or functional impairment of the meninges, brain, or vessels.^1^ These lesions may be caused by impact and/or acceleration/deceleration movement of the brain in the skull.^2^

Depending on its mechanism, TBI can be classified as closed or penetrating – which is the most common in childhood, due to falling, being run over, or suffering car accidents or aggressions.^3^ It can also be classified based on the severity of the lesion, following the Glasgow Coma Scale (GCS).^4^

In Brazil, TBI is responsible for more than 75% of child deaths.^5^ However, no recent studies on the epidemiological incidence of TBI in Brazil were found, particularly such that included children and adolescents.

TBI potentially damages the Central Nervous System (CNS) and, consequently, the central auditory pathways and the auditory cortex. Central auditory processing refers to the efficiency and effectiveness with which the Central Auditory Nervous System (CANS) uses auditory information. Acoustic signals must be adequately analyzed and interpreted to convey a meaningful message. Hence, TBI may be a risk factor for Central Auditory Processing Disorders (CAPD), which can be identified with electrophysiological and behavioral tests that assess central auditory function.^6^

However, it is difficult to assess the consequences of lesions and their meaning at different stages of development, particularly in individuals whose developmental processes are simultaneous with the effects of brain lesions. Thus, children whose brain is still developing and suffered a TBI must be evaluated, considering the complex processes that either independently or synergically influence the results of the trauma and their recovery from it.^7^, ^8^, ^9^

The literature has already given evidence of changes in auditory skills (such as speech perception in noise and integration of auditory information in dichotic activities) in adults who had milder lesions, such as concussions.^10^ Studies with adults who suffered a severe TBI also observed changes in auditory processing, demonstrated with behavioral tests.^11^

Nonetheless, few studies have addressed the central auditory pathway and TBI in children. There are scarce data on auditory processing in children in Brazil and worldwide. The authors have extensively searched the recent literature and found recent approaching cognitive functions in mild TBI, but no study involving central auditory processing assessment in children with moderate and severe TBI.

Also, some children and adolescents' particularities differ from the adults’ (e.g., greater plasticity). Hence, the consequences of acquired neurological lesions to this population's central auditory processing must be understood.

Considering the possibility that TBI may have negative consequences on the CANS, the study hypothesized that children and adolescents who suffered a TBI would perform worse in behavioral Central Auditory Processing (CAP) tests than their peers with no neurological injury.

Given the above, the objective of this study was to characterize how children and adolescents who suffered a moderate or severe TBI performed behavioral auditory processing tests and compare them with individuals without a history of TBI.

## Method

This study was carried out at the Electrophysiology and Auditory Processing Outpatient Centers of the Universidade Federal de São Paulo - UNIFESP, having been approved by the Research Ethics Committee of the Universidade Federal de São Paulo - UNIFESP, under number 0226/2014.

The study group comprised ten 8- to 18-year-old individuals of both sexes who had suffered moderate or severe closed TBI (rated 3 to 13 on the Glasgow scale at hospital admission) 3 to 24 months before they participated in the study. They were right-handed, with normal auditory thresholds, type A tympanogram, and no evident behavioral changes. All patients had been referred by the Neurosurgery and Neurotrauma Outpatient Center of the Universidade Federal de São Paulo - UNIFESP, regardless of the type of lesion, either alone or in combination. These eligibility criteria were defined by the speech-language-hearing therapists and the neurologist responsible for the study. All children's parents/guardians signed an informed consent form.

The comparison group comprised 10 individuals without a history of TBI who matched with the study group for sex, age, and grade in school. They had been referred for central auditory processing assessment.

Participants sat in a sound booth for behavioral auditory processing assessment, wearing TDH-50P supra-aural earphones. They were instructed to listen to the CD-recorded tests and then, according to each test procedure, repeat words, point to images, or imitate sounds, as requested by the examiner.

The following tests were conducted: Sound Localization Test (SLT), Sequential Memory Test for Verbal sounds (SMTV), Sequential Memory Test for Nonverbal sounds (SMTNV), Duration Pattern Test (DPT), Dichotic Consonant-Vowel Test – Free Recall condition (DCVT-FR), Dichotic Digits Test (DDT), dichotic Staggered Spondaic Word (SSW), Synthetic Sentence Identification with Ipsilateral Competing Message (SSI-ICM)/Pediatric Speech Intelligibility with Ipsilateral Competing Message (PSI-ICM), Random Gap Detection Test (RGDT), Speech Recognition Percentage Index (SRPI), and Speech by White Noise Test (SWNT). These tests encompass auditory skills and underlying physiological mechanisms that can be assessed with behavioral tests. Since the tests are selected based on chronological age, different dichotic tests were used to meet developmental aspects. The behavioral test battery used in this study is summarized in Table 1, considering auditory skills, and underlying physiological mechanisms.

**Table 1:** Behavioral tests considering auditory skill, physiological mechanism, and normal values.

Test	Auditory skill	Physiological mechanism	Normal values
SLT	Sound localization	Discriminating the direction of the sound source	4/5 correct (As long as R and L are not both wrong)
SMTV	Temporal ordering	Discriminating sequential verbal sounds	2/3 correct
SMTNV	Temporal ordering	Discriminating sequential nonverbal sounds	2/3 correct
SWNT	Auditory closure	Discriminating physically distorted sounds in monotic listening	≥ 70% and SRPI-SWNT < 20%
SSI–ICM	Figure-ground in verbal monotic listening	Discriminating overlapping sounds in monotic listening	ICM (0) ≥ 80%
			ICM (-10) ≥ 70%
			ICM (-15) ≥ 60%
DDT	Figure-ground in verbal dichotic listening	Discriminating highly predictable overlapping verbal sounds in dichotic listening	Free recall: 5 to 6 years: RE ≥ 81% and LE ≥ 74%; 7 to 8 years: RE ≥ 85% and LE ≥ 82%; > 9 years: RE = LE ≥ 95%
			Directed listening: 5 to 6 years ≥70%; 7 to 8 years ≥ 75%; > 9 years: ≥85%
SSW	Figure-ground in verbal dichotic listening	Discriminating little predictable verbal sounds in dichotic listening	≥ 90%
			Inversions ≥ 1
			Auditory effect: [-4 +4]
			Order effect: [-3 +3]
			Type A response pattern: ≥ 3
DCVT-free recall	Figure-ground in verbal dichotic listening	Discriminating overlapping verbal sounds in dichotic listening	Right-handed: ≥ 19 correct (RE > LE)
			Left-handed: ≥ 19 correct (RE > LE or LE > RE)
DPT Musical tones (up to 8 years old)	Temporal ordering	Discriminating sound patterns	3 tones: = 100% correct
			4 tones: ≥ 90% correct
DPT (Auditec) (up to 11 years old)	Temporal ordering	Discriminating sound patterns	10 and 11 years: ≥83% correct humming
			10 and 11 years: ≥76% correct naming
			≥12 years: ≥83% correct naming = humming
DPT (Musiek) (above 11 years old)	Temporal ordering	Discriminating sound patterns	10 and 11 years: ≥83% correct humming
			10 and 11 years: ≥76% correct naming
			≥12 years: ≥83% correct naming = humming
RGDT	Temporal resolution	Temporal processing	Mean ≥10 ms

SLT, Sound Localization Test; SMTV, Sequential Memory Test for Verbal sounds; SMTNV, Sequential Memory Test for Nonverbal sounds; SRPI, Speech Recognition Percentage Index; SWNT, Speech by White Noise Test; PSI, Pediatric Speech Intelligibility; SSI, Synthetic Sentence Identification; ICM, Ipsilateral Competing Message; SSW, dichotic Staggered Spondaic Word; DPT, Duration Pattern Test; N, Naming; DCVT, Dichotic Consonant-Vowel Test; RGDT, Random Gap Detection Test; ms, milliseconds.

Tests were applied in the following order: firstly, the diotic, monotic, and dichotic tests, and lastly, the temporal processing tests. All participants were analyzed considering the normal values for their age, and the tests were administered in three 1-h sessions.

Their performance in each behavioral test and the group comparisons were submitted to descriptive statistical analysis. The student's *t*-test was also used; the p-value was set at 0.05 and confidence intervals were 95%.

## Results

The study group comprised 10 children and adolescents with a mean age of 10.9 years, whose mean score on the Glasgow scale at hospital admission was 7.9 and whose mean hospital stay was 25.8 days (of which, 7.5 days were in an induced coma). The comparison was enabled by recruiting a comparison group matched for sex and age, whose participants did not have a history of TBI (Table 2).

**Table 2:** Characterization of the study group regarding sex, age, time of lesion, Glasgow scale at admission, length of stay, length of sedation, and type of lesion.

Sex	Age	Time of lesion	Glasgow at admission	Length of stay	Length of sedation	Type of lesion
M	11	10 months	3	41 days	9 days	Acute Subdural Hematoma; Left Temporal Fracture.
M	10	7 months	9	60 days	19 days	Traumatic Frontal Intraparenchymal Hematomas; Frontal Contusion.
F	9	10 months	10	33 days	10 days	Right Parietal Fracture; Right Hemisphere Edema.
M	8	11 months	13	37 days	7 days	Frontal Contusion.
F	16	12 months	4	18 days	5 days	Acute Subdural Hematoma; Right Temporoparietal Fracture.
M	8	12 months	9	10 days	0 days	Left Temporal Extradural Hematoma.
M	11	22 months	7	25 days	8 days	Left Temporal Contusion.
M	8	5 months	7	16 days	8 days	Diffuse Brain Edema.
F	18	7 months	9	8 days	3 days	Right Temporal Contusion.
F	10	18 months	8	10 days	6 days	Left Frontal Fracture; Diffuse Brain Edema.

M, Male; F, Female.

As seen in Table 2, the participants’ length of hospital stays and length of coma varied greatly, due to the different lesions – which occurred either alone or in combination, determining the severity of the condition and the necessary treatments.

Some data collected from the clinical history that might interfere with their performance (such as grades in school, failures in school, and complaints and difficulties previous to the trauma) are highlighted in Table 3. Post-trauma complaints, which were present in 100% of the sample, were also surveyed.

**Table 3:** Characterization of the sample regarding schooling level, flunking at school, difficulties before TBI, and complaints after TBI**.**

Subject	Age	Schooling level	Difficulties before TBI	Complaints after TBI
1	11	5th grade	Restlessness, inattention, and mild school difficulties.	Inattention, irritability, poor school achievement, memory change
2	10	5th grade	Inattention	Aggressiveness, irritability, inattention, school difficulties
3	16	11th grade	No	School difficulties, inattention, emotional instability, memory change
4	18	12th grade	No	Inattention and school difficulty
5	8	3rd grade	No	Irritability, important emotional instability, behavioral changes, inattention
6	10	5th grade	No	Post-TBI memory difficulties
7	10	4th grade	No	No complaints from the mother or patient
8	9	4th grade	Mild school difficulties and inattention	Much inattention, irritability, childish behavior, memory change, strong headaches, dizziness, important school difficulties
9	8	3rd grade	No	Inattention
10	8	3rd grade	No	No complaints from the mother or patient

TBI, Traumatic Brain Injury.

The results of the behavioral CAP assessment comparing both groups are given in detail in Table 4. Right- and left-ear speech by white noise test, sequential memory test (with three verbal sounds), and left-ear dichotic staggered spondaic word had statistically significant differences between the groups – the study group performed worse than the comparison group.

**Table 4:** Comparison between the performances of the comparison and study groups in the central auditory processing assessment.

			Mean	Median	Standard deviation	p-value
**SLT**		Comparison	94.0%	100%	9.7%	0.470
		Study	90.0%	100%	14.1%	
**SMTV (% correct answers)**	**3 sounds**	Comparison	100%	100%	0.0%	0.004
		Study	76.6%	66.6%	22.5%	
	**4 sounds**	Comparison	83.3%	100%	23.6%	0.754
		Study	80.0%	83.3%	23.3%	
**SMTNV (% correct answers)**	**3 sounds**	Comparison	100%	100%	0.0%	0.331
		Study	96.7%	100%	10.6%	
	**4 sounds**	Comparison	80.0%	66.6%	17.2%	0.999
		Study	80.0%	83.3%	23.3%	
**SRPI (% correct answers)**	**RE**	Comparison	91.2%	92.0%	3.2%	0.076
		Study	87.6%	88.0%	5.1%	
	**LE**	Comparison	94.0%	96.0%	3.9%	0.191
		Study	91.8%	92.0%	3.3%	
**SWNT (% correct answers)**	**RE**	Comparison	82.4%	84.0%	6.9%	< 0.001
		Study	48.4%	48.0%	14.4%	
	**LE**	Comparison	79.6%	80.0%	6.4%	< 0.001
		Study	45.6%	48.0%	19.4%	
**SSW (% correct answers)**	**RE**	Comparison	87.3%	88.8%	8.2%	0.525
		Study	82.9%	93.8%	19.7%	
	**LE**	Comparison	83.5%	85.0%	14.4%	0.040
		Study	64.3%	63.8%	23.5%	
**PSI/SSI-ICM (0) (% correct answers)**	**RE**	Comparison	100%	100%	0.0%	0.331
		Study	99.0%	100%	3.2%	
	**LE**	Comparison	98.0%	100%	6.3%	0.331
		Study	100%	100%	0.0%	
**PSI/SSI-ICM (-15) (% correct answers)**	**RE**	Comparison	85.6%	80.0%	11.3%	0.322
		Study	82.0%	80.0%	10.3%	
	**LE**	Comparison	83.3%	80.0%	8.7%	0.717
		Study	83.0%	80.0%	14.2%	
**DPT (N) (% correct answers)**		Comparison	81.2%	85.0%	17.6%	0.052
		Study	60.0%	65.0%	27.1%	
**DCVT-free recall condition (number of correct answers)**	**RE**	Comparison	12.10	12.0	2.92	0.436
		Study	11.10	10.5	2.69	
	**LE**	Comparison	7.30	7.0	1.83	0.569
		Study	6.70	6.0	2.71	
	**Errors**	Comparison	4.60	3.5	2.63	0.233
		Study	6.30	5.0	3.47	
**RGDT (ms)**		Comparison	5.63	6.0	2.39	0.134
		Study	20.25	8.8	29.36	

*t*-Student's test.

SLT, Sound Localization Test; SMTV, Sequential Memory Test for Verbal sounds; SMTNV, Sequential Memory Test for Nonverbal sounds; SRPI, Speech Recognition Percentage Index; SWNT, Speech by White Nise Test; PSI, Pediatric Speech Intelligibility; SSI, Synthetic Sentence Identification; ICM, Ipsilateral Competing Message; SSW, dichotic Staggered Spondaic Word; DPT, Duration Pattern Test; N, Naming; DCVT, Dichotic Consonant-Vowel Test; RGDT, Random Gap Detection Test; ms, milliseconds; RE, Right Ear; LE, Left Ear.

Behavioral CAP assessment results were classified as either normal or abnormal, according to the normal values of each procedure. The qualitative analysis of the study and comparison group tests are, respectively shown in Tables 5 and 6.

**Table 5:** Summary table of the qualitative analysis of the behavioral central auditory processing tests in the study group.

Green, Normal; Red, Abnormal; SLT, Sound Localization Test; SMTV, Sequential Memory Test for Verbal sounds; SMTNV, Sequential Memory Test for Nonverbal sounds; SWNT, Speech by White Noise Test; PSI, Pediatric Speech Intelligibility; SSI, Synthetic Sentence Identification; SSW, dichotic Staggered Spondaic Word; DPT, Duration Pattern Test; DCVT, Dichotic Consonant-Vowel Test; RGDT, Random Gap Detection Test.

**Table 6:** Summary table of the qualitative analysis of the behavioral central auditory processing tests in the comparison group.

Green, Normal; Red, Abnormal; SLT, Sound Localization Test; SMTV, Sequential Memory Test for Verbal sounds; SMTNV, Sequential Memory Test for Nonverbal sounds; SWNT, Speech by White Noise Test; PSI, Pediatric Speech Intelligibility; SSI, Synthetic Sentence Identification; SSW, dichotic Staggered Spondaic Word; DPT, Duration Pattern Test; DCVT, Dichotic Consonant-Vowel Test; RGDT, Random Gap Detection Test.

As seen in Table 5, some behavioral tests had abnormal results in more than 60% of the study group – such as speech by white noise (100% abnormal results), dichotic staggered spondaic word (90% abnormal results), and duration pattern test (80% abnormal results).

Table 6 classified the results of the comparison group, showing that 70% of the individuals had at least one abnormal result, whereas the other 30% had normal results in all tests.

Tables 5 and 6 indicated that no participating child or adolescent who suffered a moderate or severe TBI had normal results in the behavioral CAP assessment. All assessments resulted in at least two abnormal tests – unlike the comparison group, in which 30% of the participants had normal assessment results for their age.

**Fig. 1:**
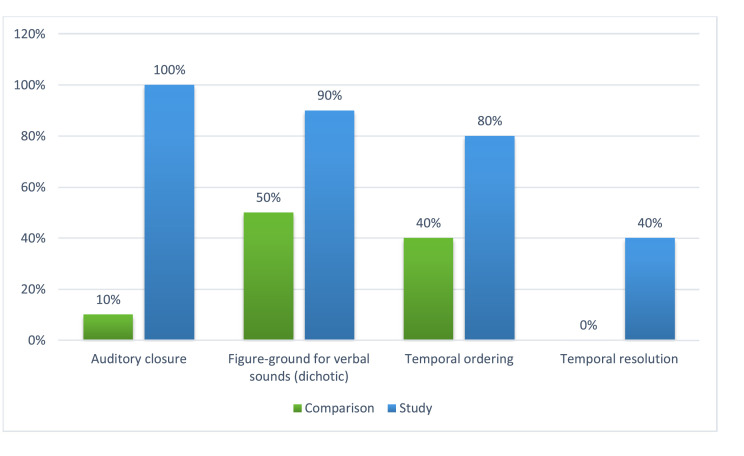
Comparison between the performances of both groups regarding the percentage of changes found in each auditory skill assessed.

Concerning auditory skills, Fig. 1 compared the performance of the two groups regarding the percentage of abnormal results. All the highlighted auditory skills reveal that the study group performed worse than the comparison group (especially in auditory closure, figure-ground in verbal dichotic listening, and temporal ordering), with statistically significant differences between the groups. There was also a difference between the groups’ performances in temporal resolution, though not statistically significant.

## Discussion

Before discussing the results, one should be aware that no previous studies with data on CAP in children after TBI were found. Therefore, research involving different populations (adults) and lesion severity (mild TBI) was used in this section to better illustrate the authors’ results.

The length of hospital stays and length of the coma of the study group participants varied greatly, which is explained by the different lesions found in them. The researchers and the neurologist responsible for the children's referrals agreed by consensus that the inclusion criteria would not be based on the type of lesion but the severity of the TBI, measured with GCS at hospital admission.

In the study group, 60% of subjects were males and 40% were females, with a mean age of 10.9 years. This corroborates the literature, which points out a predominance of TBI neurological lesions in males, occurring more often in people up to 10 years old in the municipality of São Paulo.^12^

Regardless of the type and time of lesion, all individuals had abnormal results, especially in behavioral CAP assessment. As expected, this demonstrates that TBI may affect the auditory neural substrate.

This may happen because of the susceptibility of axons to mechanical force, along with the complex connections of the auditory system and its axons, which makes the auditory system a likely place for dysfunctions due to an impact on the head. The positioning of the auditory cortex in the temporal lobe also makes it susceptible to contusions and edema.^13^

Various authors in the literature have highlighted difficulties in studying a developing CNS that has had a lesion. In both processes, plasticity results in changes in the neural substrate, reflecting behavioral changes.^7^, ^8^, ^9^

Therefore, besides the age group, some other important information was surveyed, which could interfere with the results and their interpretation (Table 3). The information revealed that the relatives of only three individuals complained of inattention and restlessness before TBI, and only two of these had previous school difficulties, classified as mild by their families. The families of the other participants had no complaints before the accident that caused TBI.

In contrast, only two families did not have post-TBI complaints. Inattention, worse school achievement, memory changes, irritability, emotional instability, aggressiveness, and dizziness were the main complaints of the majority of families.

As found in this study, the literature has demonstrated in clinical studies with children who suffered a TBI that residual problems may occur in various abilities, including intellectual capacity, attention, memory, and even psychiatric disorders.^7^^,^^14^^,^^15^

Table 4 compares the groups’ performances in the behavioral CAP tests. It shows a statistically significant difference between them in the speech by white noise test and dichotic staggered spondaic word.

While both groups had similar performances in the word recognition score, there was an important decrease in the percentage of correct answers in the speech by white noise test in both ears in the study group. A decrease of up to 20% in the number of correct answers is expected, which highlights the degraded performance when noise is introduced, using the same stimuli previously presented in silence.

As for the dichotic staggered spondaic word, a difference was observed between the ears in the study group (the left ear performed worse), statistically different from the comparison group. This interaural difference may significantly impair the person's performance in noisy environments, indicating the need for a specific intervention to attenuate the difference. An interaural difference in speech perception tests with difficult hearing may significantly impair communicative performance, with consequences to learning as well.

There was a statistically significant difference between the groups in the sequential memory test for verbal sounds, although both groups’ percentage of correct answers is compatible with normal values for this test.

The dichotic consonant-vowel test revealed that the study group had advantages in the right ear, indicating left-hemisphere dominance. According to the hemisphere specialization theory, the left temporal lobe plays a more important role than the right one in perceiving linguistic stimuli. This suggests that when different verbal stimuli are presented to both ears, the stimuli that reach the ear opposite to the dominant hemisphere are more efficiently recognized.^16^ Thus, left-hemisphere dominance in study group children and adolescents suggests a good prognosis, as the dominant auditory pathway for verbal stimuli still had an advantage, despite the lesions. The same hemisphere dominance was perceived in the comparison group, as expected.

Some of the tests had abnormal results for most of the children and adolescents in the study group. It was the case of speech by white noise (100% abnormal results), dichotic staggered spondaic word (90% abnormal results), and duration pattern test (80% abnormal results). These are the tests with the greatest potential to detect changes in these populations’ CAP (Table 5).

In a study with adults who suffered a severe TBI, the duration pattern test had the highest index of abnormal results (60%), while random gap detection had 50% and the dichotic consonant-vowel test had 40% abnormal results.^17^ It can be thus suggested that, in children and adults who suffered a moderate or severe TBI, behavioral CAP assessments were quite important to detect changes, although the tests with the most abnormal results were different in each population.

Children who had concussions were also studied, and their results showed changes in speech perception of noise, in comparison with their peers without a previous history of concussion.^18^

A study assessed post-TBI CAP in 62 children and showed that 16% of those admitted to the rehabilitation unit after suffering a moderate or severe TBI had a poor performance in CAP tests involving low-redundancy speech^19^ – which corroborates the findings in the present study regarding the speech by white noise test.

In the present research, the behavioral tests with abnormal results in the study group corresponded to auditory closure, figure-ground in verbal dichotic listening, and temporal ordering (Fig. 1). They predict difficulties in assigning meaning to auditory information, particularly in analyzing the phonemic system of language, suprasegmental features of speech, and organization of sound events in time. This coincides with the main complaints of the children's and adolescents’ relatives, which included inattention, memory difficulties, and poor school achievement.

As seen in Table 5, all participants in the study group had abnormal results in at least two behavioral CAP assessment tests – one of which was the speech by white noise test, whose results were abnormal in all children and adolescents.

The speech by white noise test assesses auditory closure. Considering that all participants were schoolchildren, auditory closure impairment may hinder the attention and concentration necessary for effective learning, as the school, particularly the classroom, is a rather noisy environment.

Another important point to highlight is that temporal processing, assessed with the duration pattern test in this study, had abnormal results in 80% of the people in the study group. Discriminating duration patterns and sound frequencies and perceiving temporal aspects of sound are known to play an essential role in speech perception, speech sound segmentation, and language learning and comprehension, either spoken or written.

These results demonstrated that CAPD was detected in all study group participants, assessed with behavioral CAP tests. As for the comparison group (Table 6), 40% of the participants had normal results, and 20% had abnormal results in only one test, which does not characterize CAPD. Therefore, 60% of the participants in the comparison group had normal results in the CAP assessment.

It is also important to highlight that the CAP tests with the most abnormal results in the comparison group were different from those in the study group. This demonstrates that CAPD may occur in the two populations, although with different profiles of changes.

Such results indicate that CAP assessments should be part of the process of evaluating TBI sequelae, particularly in schoolchildren. Likewise, electrophysiological assessments of hearing should be included, considering the academic and language developments that may result from CAP changes.

A limitation of this study was the difficulty in recruiting children per type of lesion and forming groups with similar neurological injuries to understand how CAP behaves in each case. Even though the findings cannot be generalized due to the small sample size, they point out neuroradiologic issues that go beyond the audiogram.^20^

Further studies are needed to better understand CAP changes caused by neurological lesions and form groups as homogeneous as possible regarding the characteristics of the lesion, age at injury, and other factors that may influence their performance. This will help understand the consequences of such lesions to CAP, leading to the implementation of adequate rehabilitation programs that consider the development and improvement of auditory skills – which are necessary for good speech and language development.

## Conclusion

Central auditory processing disorders were identified in all study group participants, with statistically significant differences in auditory closure and temporal processing in comparison with the control group.

## Authors' contributions

The authors contributed sufficiently to the paper and take public responsibility for their respective parts of the content.

## Declaration of Competing Interest

The authors declare no conflicts of interest.
